# Homozygous Mutation in the Insulin Receptor Gene Associated with Mild Type A Insulin Resistance Syndrome: A Case Report

**DOI:** 10.4274/jcrpe.galenos.2020.2019.0213

**Published:** 2021-02-26

**Authors:** Bülent Hacihamdioğlu, Elif Gülşah Baş, Kenan Delil

**Affiliations:** 1 İstinye University Faculty of Medicine, Department of Pediatric Endocrinology, İstanbul, Turkey; 2 Bahçeşehir University Faculty of Medicine, İstanbul, Turkey; 3 Marmara University Faculty of Medicine, Department of Medical Genetics, İstanbul, Turkey

**Keywords:** Hirsutism, insulin resistance, insulin receptor gene

## Abstract

Insulin receptor (INSR) mutations lead to heterogeneous disorders that may be as severe as Donohue syndrome or as mild as “type A insulin resistance syndrome”. Patients with severe disorders usually harbor homozygous or compound heterozygous mutations. In contrast, type A insulin resistance syndrome has been associated with heterozygous mutations; homozygous mutations are rarely responsible for this condition. We report a novel, homozygous mutation, p.Leu260Arg in exon 3, of the *INSR* gene in a female adolescent patient with type A insulin resistance syndrome together with clinical details of her medical follow-up. Different mutations in the *INSR* gene cause different phenotype and vary depending on the inheritance pattern. This report adds to the literature, increases understanding of the disease mechanism and aids in genetic counseling.

**What is already known on this topic?**

Insulin receptor (INSR) mutations lead to heterogeneous disorders that may be as severe as Donohue syndrome or as mild as “type A insulin resistance syndrome”. Patients with severe disorders usually harbor homozygous or compound heterozygous mutations whereas type A insulin resistance syndrome is usually associated with a heterozygous mutations. Homozygous *INSR* gene mutations may rarely be responsible for mild type insulin resistance syndrome.

**What this study adds?**

The case presented with mild type A insulin resistance syndrome, was due to a novel homozygous mutation in the *INSR* gene. The novel mutation was p.Leu260Arg in exon 3 of the *INSR* gene. This highlights that homozygous *INSR* mutations may also cause mild clinical forms.

## Introduction

A functional insulin receptor (INSR) is crucial for eliciting the intracellular molecular effects of insulin and *INSR* mutations lead to genetically severe insulin resistance. *INSR* is a transmembrane protein and a member of the receptor tyrosine kinase family. This receptor is a heterotetramer, consisting of two α and two β subunits. The α subunits are extracellular, whereas the β subunits extend from the extracellular side of the membrane, traverse the membrane and protrude into the cytoplasm, this latter region possesses the tyrosine kinase activity. Activation of *INSR* requires trans-autophosphorylation of one β subunit by the other β subunit. A single gene,* INSR*, encodes for both subunits, and is located on chromosome 19. Each allele of this gene encodes one αβ half-receptor and two of them form αββα heterotetrameric *INSR*. This phenomenon explains how heterozygous mutations may result in impaired β subunit tyrosine kinase activity (1,2). 

INSR mutations lead to heterogeneous disorders that range in severity from Donohue syndrome (leprechaunism) and Rabson-Mendenhall syndrome to the mild “type A insulin resistance syndrome”. Patients with severe disorders are usually homozygous or compound heterozygous for these mutations (3,4). In contrast, type A insulin resistance syndrome has been associated with heterozygous mutations; homozygous mutations are rarely responsible for this condition (5,6,7).

Type A insulin resistance syndrome manifests itself in the peripubertal period, as oligomenorrhea and hyperandrogenism with acanthosis nigricans. In this article, we report an adolescent with type A insulin resistance syndrome due to a novel homozygous mutation in the *INSR* gene and also report details of three years of medical follow-up.

## Case Report

**Figure 1:**
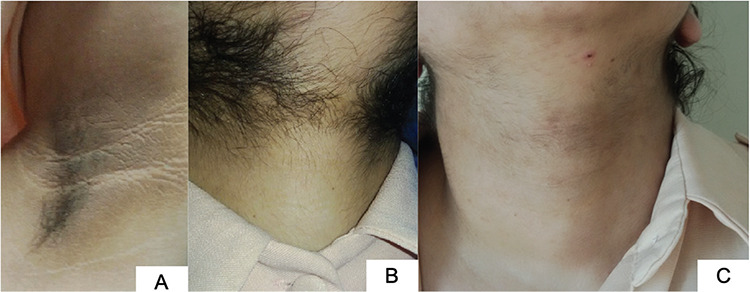
Clinical features of patient. A) severe acanthosis nigricans; B) hirsutism before treatment; and C) after treatment

A 12-year-old girl was admitted to the pediatric endocrinology department for excess hair growing on her body. This complaint had become evident over the preceding year. The patient was born of non-consanguineous marriage and her parents were healthy. She was born with a normal weight and her past medical history was uneventful. She had a severe hirsutism (Modified Ferriman-Gallwey Score was 30), acneiform rash on her face and severe acanthosis nigricans was observed in the axilla, neck and antecubital area (Figure 1A, 1B). Her blood pressure was 110-70 mmHg. Her height was 156.5 cm (70^th^ percentile), weight was 68.4 kg (99^th ^percentile), and body mass index was 27.9 kg/m^2^ (98^th^ percentile) at the admission.

**Figure 2:**
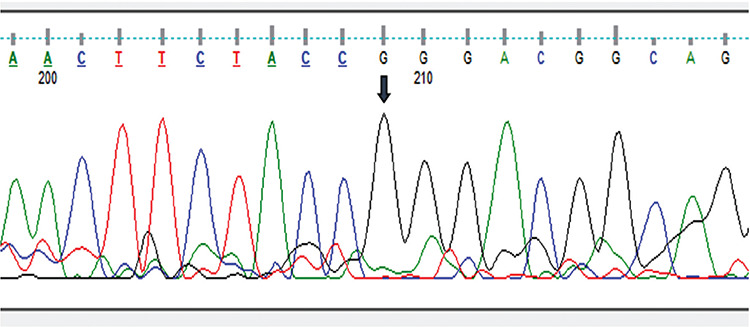
DNA sequencing chromatogram of the patient. The arrow indicates a homozygous *NM_000208.4 c.779 T>G (p.Leu260Arg)* mutation in exon 3 of the *INSR* gene

Her pubertal development was Tanner stage 4 and her bone age was 13.5 years old. There was no history of spontaneous menarche. Her laboratory examinations results are detailed in Table 1. Laboratory investigations revealed elevated fasting insulin level with normal fasting glucose. Glycohemoglobin (HbA1c) level and oral glucose tolerance test results showed that she was in a pre-diabetic state with marked hyperinsulinemia. Despite the severe hyperinsulinemia, she had normal triglyceride, high-density lipoprotein (HDL) cholesterol and sex hormone binding globulin (SHBG) level and there was no hepatosteatosis on ultrasonographic evaluation. Her gonadotropin levels revealed luteinizing hormone dominancy with increased testosterone level. There was a polycystic ovary appearance on ultrasonographic evaluation. Through the findings of clinical and laboratory examination, type A insulin resistance syndrome was considered and *INSR* mutation analyses were planned. DNA Sanger Sequence analyses of all coding exons of *INSR* showed that a novel, homozygous mutation, *NM_000208.4 c.779 T>G *(p.Leu260Arg) was present in exon 3 (Figure 2). Genetic analysis of the parents demonstrated both were carriers of the same mutation. There was no clinical or biochemical hyperandrogenism or disorder of glucose metabolism in her mother. Unfortunately, her father was not investigated due to extenuating circumstances.

First choice treatment was metformin with life style modification. It was planned to increase the dose gradually up to 2 gram/day. Unfortunately the patient was unable to comply with the life style modification consistently during whole therapy process. After one year of this therapy, oral contraceptive (OCP) (OCP; 2 mg cyproterone acetate plus 35 microgram ethinyl estradiol) were added because of increasing hirsutism. The clinical aim of this treatment was to suppress ovarian hyperandrogenism and to gain the additional benefit of the anti-androgenic potential of cyproterone acetate. After one year starting OCP her hirsutism score had markedly decreased (Figure 1C). Menarche also occurred after this treatment.

During her clinical follow-up basal-bolus insulin regimen was added into her therapy because of marked hyperglycemia, especially in the postprandial period, and a high HbA1c level (8.6%). Her HbA1c decreased to 7% after six months of basal-bolus insulin treatment. During the follow-up, bolus insulin was discontinued while retaining basal insulin and metformin therapy. The mean HbA1c was 7.4% at one-year follow-up. The patient and her mother provided informed consent.

Written consent form was obtained from the parents.

## Discussion

If a patient presents in the adolescent period with hyperandrogenism and severe insulin resistance, and these findings are not explained by other reasons, such as obesity, then investigating clinicians should consider genetic insulin resistance syndromes. Mutations of *INSR* should be kept in mind in patients with severe insulin resistance but without metabolic dyslipidemia, low SHBG level and hepatosteatosis. Clinical and laboratory features of this patient were suggestive of *INSR* mutation.

Metabolic dyslipidemia (hypertriglyceridemia and low HDL-cholesterol levels) and steatohepatitis are closely associated with prevalent forms of insulin resistance (8). A key factor in the development of metabolic dyslipidemia and hepatic steatosis is postreceptor hepatic insulin resistance. Reduced liver fat synthesis plays a key role protection from dyslipidemia observed in patients with insulin receptoropathy (9). Absence of metabolic dyslipidemia and fatty liver in a patient with severe insulin resistance, as in this patient, is suggestive of a primary *INSR* mutation.

There are no obvious genotype–phenotype correlations for *INSR* mutations. It has been suggested that the homozygous mutations of the a-subunit cause more severe clinical features, whereas heterozygous b-subunit mutations lead to milder forms (1,3). However, this is not the case in all patients. Five patients type A insulin resistance patients with a-subunit mutations have been reported, similar to this case (5,6,7). Generally, patients with type A insulin resistance syndrome have been found to have heterozygous mutations. However, homozygous inheritance of mutations may rarely be responsible for this disease. Nakashima et al (7) previously described a Japanese patient, diagnosed as type A insulin resistance syndrome with a homozygous mutation. Later, the same mutation was detected in a patient from Morocco (6). Homozygous inheritance was reported in three of eight patients with type A resistance syndrome in another series (6). Interestingly all type A insulin resistance syndrome patients with a subunit mutations had a homozygous inheritance pattern (6). We detected a novel homozygous mutation in exon 3 at position 260 (p.Leu260Arg). A homozygous mutation that resulted in proline instead of leucine at the same codon was reported previously in a family. The homozygous form of this specific variant, (p.Leu260Pro), has been associated with leprechaunism (10). It was concluded that a mutation in this region disrupts the signaling from the insulin binding site on the a-subunit with the tyrosine kinase domain on the b subunit (10). It was reported that the heterozygous form of p.Leu260Pro was associated with a normal phenotype with mild insulin resistance (1). It has also been reported that different missense mutations in the same codon can cause variable phenotype (3).

Currently available therapies for genetic insulin resistance syndromes are nonspecific. Dietary changes and exercise, in addition to drug therapy (metformin with or without insulin), have been the mainstay with the clinical aim of reducing insulin resistance (8). Commonly, patients with mild *INSR* defects present peripubertally with oligomenorrhea and hyperandrogenism and acanthosis nigricans; misdiagnosis with polycystic ovary syndrome has occurred (11). At presentation, diabetic hyperglycemia has often yet to develop, as in our patient (8). Hirsutism treatment may be difficult in these cases, but a good response to cyproterone acetate treatment has been described (11).

## Conclusion

In conclusion, we report a novel, homozygous mutation, p.Leu260Arg, in exon 3 of the *INSR* gene in a patient with type A insulin resistance syndrome. The unusual feature in this case is the homozygous inheritance pattern. As different mutations in the *INSR* gene cause different phenotypes, as do different inheritance patterns, this report is important for expanding understanding of the disease mechanisms at work and in aiding the genetic counseling process. It should be borne in mind that homozygous *INSR* mutations might, rarely, be associated with type A insulin resistance.

## Figures and Tables

**Table 1:**
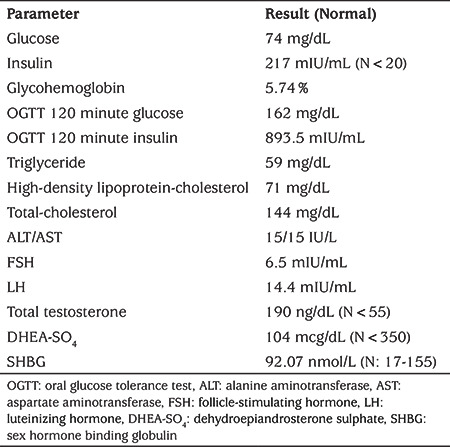
Laboratory data of a patient
